# *Toxoplasma gondii* in sympatric wild herbivores and carnivores: epidemiology of infection in the Western Alps

**DOI:** 10.1186/1756-3305-7-196

**Published:** 2014-04-24

**Authors:** Ezio Ferroglio, Fabio Bosio, Anna Trisciuoglio, Stefania Zanet

**Affiliations:** 1 Dipartimento di Scienze Veterinarie, Università degli Studi di Torino, via L. da Vinci 44 10095, Grugliasco, (TO), Italy

**Keywords:** *Toxoplasma gondii*, PCR, ELISA, Wildlife, Livestock, Italy

## Abstract

**Background:**

*Toxoplasma gondii* is an apicomplexan parasite that is able to infect almost all warm blooded animals. In Europe, the domestic cat is the main definitive host. Worldwide, 6 billion people are infected with this parasite. The goal of our research is to evaluate the prevalence of *T. gondii* infection in wild animals from a previously unsampled area in Northern Italy where 0.1% of women seroconvert during pregnancy each year.

**Methods:**

We sampled and tested skeletal muscle and central nervous system tissue of 355 wild animals by PCR (n = 121 roe deer *Capreolus capreolus*, n = 105 wild boar *Sus scrofa*, n = 94 red fox *Vulpes vulpes*, n = 22 alpine chamois *Rupicapra rupicapra*, n = 13 red deer *Cervus elaphus*).

**Results:**

The overall prevalence of infection with *T. gondii* was 10.99% (confidence interval (CI) 95% 8.14%-14.67%). A higher rate of infection was recorded in carnivores and omnivores (red fox 20.21%, CI 95% 13.34%-29.43%; wild boar 16.19%, CI 95% 10.36%-24.41%) compared to ruminants (2.48%, CI 95% 0.85%-7.04% in roe deer; 0.00%, CI 95% 0.00%-22.81% in red deer, and 0.00% alpine chamois (CI 95% 0.00%-14.87%) confirming the importance of tissue cysts in transmitting infection.

**Conclusions:**

The relatively high prevalence of *T. gondii* DNA in highly consumed game species (wild boar and roe deer) gives valuable insights into *T. gondii* epidemiology and may contribute to improve prevention and control of foodborne toxoplasmosis in humans.

## Background

*Toxoplasma gondii* is an apicomplexan parasite that infects a wide variety of warm-blooded animals with an asexual stage in intermediate hosts and a sexual stage in a definitive host, which may be any species of domestic or wild felids [1]. *T. gondii* zoonotic infection is present worldwide. The study by Torgerson and Macphearson, [2] reviewed global seroprevalence values in women of childbearing age that are reported to range from 6% to 80%. In humans infection can be congenital or can be acquired postnatally by ingesting tissue cysts from undercooked meat, or by consuming food/drink contaminated with oocysts [3,4]. In the study area (Piedmont Region, Italy) seroprevalence in women of childbearing age is 21.5% [5] and the incidence of infection during pregnancy is 0.1% [6]. Raw or cured meat products, especially pork, mutton and wild game, are the principal sources of infection [7-10]. Game meat is traditionally consumed in Northern Italy (4 kg/year per capita in hunters families) [11] with an increasing trend linked to the growth of wild ungulate populations [12,13]. In 2011, 1600 chamois, 3460 roe deer, 611 red deer and more than 10,000 wild boar were harvested during hunting season [14]. Considering the abundance of big game species in the study area and the zoonotic burden of toxoplasmosis, we decided to assess *T. gondii* prevalence within a previously unsampled area in Northern Italy.

## Methods

All tested animals were sampled within the Piedmont Region (Northwestern Italy) from an overall area of 20,000 km^2^. A portion of skeletal muscle was collected from 355 wild ungulates and carnivores hunted or accidentally found dead between October 2009 and December 2012 (ethical approval to the project was given by Dept. Veterinary Sciences, University of Turin). From 213, it was also possible to sample and test a portion of the central nervous system (CNS) of these animals (Table 1). Five wild species were sampled: roe deer *Capreolus capreolus* (n = 121), red deer *Cervus elaphus* (n = 13), alpine chamois *Rupicapra rupicapra* (n = 22), wild boar *Sus scrofa* (n = 105), and red fox *Vulpes vulpes* (n = 94) (Table 1). Each sample was individually stored at -20°C until further processing.

a. *Molecular detection of T. gondii: sample processing and PCR analysis*

Total genomic DNA was extracted from ≈ 25 mg of skeletal muscle and from ≈ 25 mg of CNS homogenate, using the commercial GenElute^®^ Mammalian Genome DNA Miniprep (Sigma-Aldrich, St. Louis, MO, USA). Extracted DNA was quantified using NanoDrop 2000 (NanoDrop Technologies, Montchanin, DE, USA). PCR targeted a 200–300 fold repetitive 529 bp DNA fragment using as primers TOX4 (5′-CGCTGCAGGGAGGAAGACGAAAGTTG-3′) and TOX5 (5′-CGCTGCAGACACAGTGCATCTGGATT-3′) [15]. The PCR reaction mixture (25 μl) contained ≈ 100 ng of DNA template, 2.5 μl 10X PCR buffer, 5 μl of Q Buffer, 2.5 UI of HotStarTaq DNA Polymerase (Qiagen, Milan, Italy), 0.5 μl of dNTPs mix (10 mM of each dNTP, Sigma-Aldrich, St. Louis, MO, USA), and 12.5 pmol of each primer. An initial denaturation step of 15 min at 95°C was followed by 35 repeats of 1 min at 94°C, 1 min at 55°C, and 1 min at 72°C, and a final elongation step of 10 min at 72°C. PCR amplicons were visualized on a 2% agarose gel, with a UV transilluminator (GelDoc 1000, Bio-Rad, Hercules, CA). All PCR positive am plicons were purified (Nucleospin Extract II kit, Macherey-Nagel, Düren, Germany) and directly sequenced (Macrogen Europe, The Netherlands) to confirm PCR results. All standard precautions were taken to minimize the risk of cross-contamination (PCR preparation and addition of DNA was carried out in separate laminar-flow cabinets using DNA-free disposable material. Positive and negative controls were processed in parallel to all samples).

b. *Statistical analysis*

R software 3.0.1 [16] was used for statistical analysis. Risk factors were assessed separately for each species. We considered the potential risk factors for *T. gondii* infection as: species, sex, age and the year of sampling. To compare the performance of PCR on skeletal muscle and on CNS we used the Mc Nemar’s test for paired data.

**Table 1:** PCR results on skeletal muscle and CNS of tested wildlife species

**Species**	**Skeletal muscle**		**CNS**		**Total**	
	**PCR pos/total**	**Prevalence [CI 95%]**	**PCR pos/total**	**Prevalence [CI 95%]**	**Total pos/total**	**Prevalence [CI 95%]**
Roe deer	3/121	2.48% [0.85 -7.04]	0/72	0% [0–5.07]	3/121	2.48% [0.85 -7.04]
Wild boar	17/105	16.19% [10.36 - 24.41]	0/60	0% [0–6.02]	17/105	16.19% [10.36 - 24.41]
Red fox	15/94	15.96% [9.92 - 24.67]	5/81	6.17% [2.67 - 13.65]	19/94	20.21% [13.34 - 29.43]
Alpine chamois	0/22	0% [0–14.87]	0/0	n.d.	0/22	0% [0–14.87]
Red deer	0/13	0% [0–22.81]	0/0	n.d.	0/13	0% [0–22.81]
**Total**	**35/355**	**9.86 [7.17 - 13.4]**	**5/213**	**2.35% [1.01 - 5.38]**	**39/355**	**10.99% [8.14 - 14.67]**

Skeletal muscle and CNS were tested in parallel by PCR for *T. gondii*. PCR results obtained on each tissue type are reported separately for each of the tested animal species.

## Results and discussion

The overall prevalence of infection recorded in the wild species tested in Northwestern Italy was 10.99% [39/355] (CI 95%, 8.14%–14.67%). PCR on skeletal muscle (p = 9.86%; CI 95%, 7.17% - 13.4%) resulted significantly more sensible (Χ^*2*^ = 11.11, p < 0.005) in detecting *T. gondii* DNA than on CNS (p = 2.35%; CI 95%, 1.01% - 5.38%). Nevertheless, testing CNS allowed us to detect 4 positive animals that tested negative by PCR on muscle. The highest prevalence was recorded in red fox (p = 20.21%; CI 95%, 13.34%-29.43%) followed by wild boar (p = 16.19%; CI 95%, 10.36%-24.41%) and roe deer (p = 2.48%; CI 95%, 0.85%-7.04%). None of the red deer (p = 0%; CI 95%, 0%-22.81%) or chamois (p = 0%; CI 95%, 0%-14.87%) tested positive (Table 1). The prevalence of *T. gondii* infection in red fox (Odds Ratio = 3.05; Χ^*2*^ = 11.13; p < 0.001) and in wild boar (Odds Ratio = 2.0; Χ^*2*^ = 4.13; p < 0.05) was significantly higher than in the other tested species but no significant difference existed between the two species (Odds ratio = 1.311; Χ^*2*^ = 0.54; p > 0.1). The relatively high prevalence (p = 10.99%) of *T. gondii* recorded in Piedmont evidenced a widespread presence of the parasite in wildlife although relevant discrepancies exist among the tested species. The parasite was absent or was found at very low prevalence in ruminants: red deer and chamois (0%), roe deer (2.48%), while higher prevalence of infection was recorded in omnivores (wild boar p = 16.19%) and carnivores (red fox p = 20.21%) respectively. These findings confirm what Smith and Frenkel [1] described for North America, where increasingly higher prevalence of infection was found in herbivores (9%), omnivores (21%) and carnivores (52%). This reflects the higher probability of a carnivore or omnivore to consume tissues infected with *T. gondii* than the probability of a herbivore to ingest *T. gondii* oocysts from the environment. This is especially true in epidemiological contexts where there is only one species acting as definitive host (in the studied area it is the domestic cat) and contributing to oocyst dissemination [1]. Prevalence in red fox ranged from 14% in Germany [17] to 16.1% in Central Italy [18], 18.8% in Belgium [19] and 68% in Hungary [20]. PCR results on wild boar from France recorded a prevalence of 14.19% [21], while 5% of Belgian roe deer and 0% of red deer were positive by PCR [19]. The absence or low prevalence of infection in alpine chamois and red deer is consistent with previous data [22,23]. In the study area the alpine chamois has an altitudinal range between 600 and 2700 m a.s.l. In the high altitude alpine area occupied by chamois and red deer there are no major urban settlements nor anthropic areas hence domestic cats are uncommon and infection with sporulated oocysts is unlikely to occur. On the contrary roe deer is a ubiquitous and more synanthropic species, which is more likely to encounter oocysts. In *Sylvilagus floridanus* from the same geographical area, *T. gondii* was recorded with a prevalence of 2.08% [24]. *S. floridanus* is as ubiquitous as roe deer, and feeds exclusively from the ground. For both *C. capreolus* and *S. floridanus* tissue cyst consumption can be ruled out, and their similar infection rates (2.48% and 2.08%) can be ascribed to consumption of oocysts eliminated by cats into the environment. Both surface water [25,26] as well municipal drinking water [27] can be highly contaminated, and are recognized as among the major sources of infection [25,26]. Future genotyping by PCR-RFLP [28,29] of *T. gondii* isolates will allow a clearer understanding of the role of wildlife in *T. gondii* epidemiology as a recent work by Wendte et al. [30] evidenced that, beside the widespread genotype II, wildlife hosts several recombinant genotypes.

## Conclusions

Economic, social and bioclimatic changes are causing ever-increasing contact among wildlife, humans and domestic animals [31] and the role of wildlife as a source of zoonotic diseases should be specially monitored [32]. Game meat consumption is steadily increasing [12] and the presence of *T. gondii* DNA in skeletal muscles of 16.19% of wild boar and 2.48% of roe deer could indicate a possible source of human infection. Wildlife can become a valuable indicator of environmental contamination with *T. gondii* oocysts.

## Competing interests

The authors declare they have no competing interests.

## Authors’ contributions

EF coordinated sample collection and testing, FB and AT performed the experiments and analyzed data, SZ drafted the manuscript. All authors read and approved the final version of the manuscript.
